# Hippo pathway in cell–cell communication: emerging roles in development and regeneration

**DOI:** 10.1186/s41232-024-00331-8

**Published:** 2024-04-02

**Authors:** Akihiro Nita, Toshiro Moroishi

**Affiliations:** 1 Department of Molecular and Medical Pharmacology, Faculty of Life Sciences, Kumamoto University, 1-1-1 Honjo, Kumamoto, 860-8556 Japan; 2 Center for Metabolic Regulation of Healthy Aging, Faculty of Life Sciences, Kumamoto University, Kumamoto, Japan

**Keywords:** The Hippo pathway, Cell–cell communication, Development, Regeneration, Heterogeneity

## Abstract

The Hippo pathway is a central regulator of tissue growth that has been widely studied in mammalian organ development, regeneration, and cancer biology. Although previous studies have convincingly revealed its cell-autonomous functions in controlling cell fate, such as cell proliferation, survival, and differentiation, accumulating evidence in recent years has revealed its non-cell-autonomous functions. This pathway regulates cell–cell communication through direct interactions, soluble factors, extracellular vesicles, and the extracellular matrix, providing a range of options for controlling diverse biological processes. Consequently, the Hippo pathway not only dictates the fate of individual cells but also triggers multicellular responses involving both tissue-resident cells and infiltrating immune cells. Here, we have highlighted the recent understanding of the molecular mechanisms by which the Hippo pathway controls cell–cell communication and discuss its importance in tissue homeostasis, especially in development and regeneration.

## Background

The Hippo pathway was first discovered through genetic mosaic screening aimed at identifying mutant tumor suppressors in *Drosophila melanogaster*. In these screenings, it was observed that mutations causing loss of function in Hippo pathway components resulted in significant organ overgrowth, owing to enhanced cell proliferation and diminished cell death [1]. The discovery that this pathway, along with its involvement in cell proliferation, is preserved in mammals evoked high expectations and has garnered significant interest in recent years. Experimental evidence has consistently demonstrated that the Hippo pathway plays a substantial role in various aspects of cancer progression and has crucial regulatory functions in organ development, regeneration, and stem cell biology [2–4].

Recently, the complexity of the Hippo pathway has expanded substantially with the identification of its non-cell-autonomous functions and accumulating evidence suggesting that the Hippo pathway influences certain aspects of physiological functions by regulating cell–cell communication. This highlights that the field is moving away from the concept of a straightforward intracellular signaling pathway to a view in which the Hippo pathway is a crucial regulator of intercellular communication and a central hub within the cellular network of multicellular organisms. In this review, we summarized the recent understanding of the mechanisms by which the Hippo pathway modulates the surrounding environment through cell–cell communication and its role in tissue physiology and pathology, especially in the context of development and regeneration. The foundational framework of the Hippo pathway was initially outlined in the *Drosophila*. Subsequent studies in flies and mammals have significantly broadened our understanding of the Hippo pathway as a conserved signaling pathway controlling cell growth and fate [5]. Although the basic structure of the Hippo pathway has been evolutionarily preserved, recent findings indicate substantial variation in regulatory mechanisms and functions between flies and mammals. This review primarily delves into contemporary insights into the role of the Hippo pathway in cell–cell communication within mammalian systems.

## Main text

### Complex roles of the Hippo pathway in multicellular organisms

#### The Hippo pathway

**Fig. 1:**
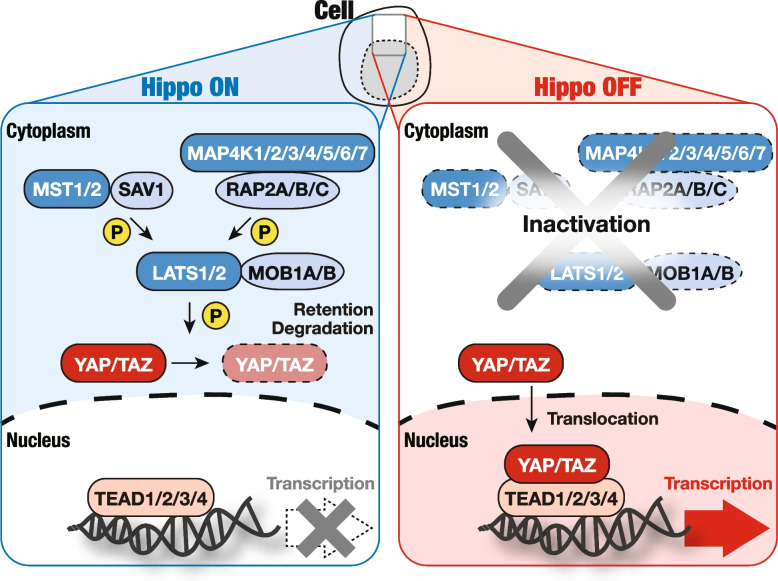
Molecular functions of the Hippo pathway. The core of the Hippo pathway consists of a kinase cascade, including MST1/2, MAP4K1/2/3/4/5/6/7, and LATS1/2, along with their binding partners SAV1, RAP2A/B/C, and MOB1A/B. When the Hippo pathway is activated, the upstream kinases MST1/2 and MAP4K1/2/3/4/5/6/7 induce the phosphorylation and activation of LATS1/2 kinases. LATS1/2 then phosphorylates the downstream transcriptional co-activators YAP/TAZ, inhibiting their functions by promoting their cytoplasmic retention and subsequent degradation. When the Hippo pathway is deactivated, unphosphorylated YAP/TAZ translocate to the nucleus, whereby they promote the transcriptional activity of the transcription factors TEAD1/2/3/4 to regulate the transcription of genes associated with cell-fate decision, such as cell proliferation, survival, and differentiation

More than 30 components of the Hippo pathway have been identified since its discovery [6–10]. The key components—mammalian STE20-like kinase 1/2 (MST1/2; Hippo in *Drosophila*) [7, 9, 10], mitogen-activated protein kinase kinase kinase kinase 1/2/3/5 (MAP4K1/2/3/5; Happyhour in *Drosophila*) [11], MAP4K4/6/7 (Misshapen in *Drosophila*) [12], large tumor suppressor 1/2 (LATS1/2; Warts in *Drosophila*) [1, 13–15], Salvador family WW domain containing protein 1 (SAV1; Salvador in *Drosophila*) [6, 8–10, 15], MOB kinase activator 1A/B (MOB1A/B; mats in *Drosophila*) [16], Yes-associated protein (YAP) and transcriptional coactivator with PDZ-binding motif (TAZ) (Yorkie in *Drosophila*) [17], and TEA domain transcriptional factor 1/2/3/4 (TEAD1/2/3/4; scalloped in *Drosophila*) [18–20]—were discovered through genetic studies involving flies or mice, as well as cell biology studies using human cell lines conducted between 1995 and 2015. Molecular function of the mammalian Hippo pathway is an intracellular phosphorylation-dependent signaling cascade; when the upstream signals, including soluble factors, mechanosignals, and metabolic and nutrient signals, activate the core LATS1/2 kinases through the upstream MST1/2 or MAP4K1/2/3/4/5/6/7 kinases, LATS1/2 phosphorylate and inhibit the transcriptional co-activators YAP/TAZ, thereby suppressing target gene transcription. Conversely, when LATS1/2 is inactivated, unphosphorylated YAP/TAZ translocate into the nucleus, where they bind and activate mainly TEAD transcription factors (Fig. 1). Therefore, the functional output of the Hippo pathway is the activation of LATS1/2 kinases and the subsequent inhibition of YAP/TAZ transcriptional co-activators, suppressing gene expression. For a more in-depth understanding of the mechanistic regulation of the Hippo pathway, one can refer to other reviews [21–24].

Biological functions of the Hippo pathway have been studied in genetically engineered animal models in which components of this pathway have been deleted or overexpressed [23]. Previous studies in flies and mice have shown that inactivation of upstream kinase components or activation of downstream transcriptional components can result in tissue overgrowth [6, 9, 10, 15, 25–29]. For instance, inducing liver-specific YAP overexpression in mice results in notable enlargement of the liver. Upon the cessation of YAP overexpression, the liver returns to its original size through apoptosis [25, 30]. Similarly, deletion of the upstream kinase MST1/2 in the liver results in hepatomegaly and eventual tumor formation [26]. Therefore, it is generally accepted that the Hippo pathway negatively regulates organ growth [5, 30–34].

Although most studies support this concept, recent studies have revealed that the experimental setting may affect the tissue phenotype induced by Hippo mutations. For example, young *Lats1*^−/−^/*Lats2*^F/F^ mice injected with Cre-expressing adenovirus developed massive hepatomegaly due to overproliferation of cytokeratin-positive biliary epithelial cells (BECs) [35, 36]. In contrast, in *albumin*-Cre/*Lats1*^F/F^/*Lats2*^F/F^ mice, LATS1/2 deletion is induced during the embryonic stage. BECs in these animals exhibit excessive proliferation, and hepatoblasts fail to mature into hepatocytes, leading to liver dysfunction and perinatal mortality [37]. Therefore, the effects of LATS1/2 deletion on tissue growth vary depending on the timing and method of gene deletion, adding an additional level of complexity to our understanding of the Hippo pathway during organ growth.

In addition, the Hippo pathway is coordinately regulated by multiple components, and the deletion of different components sometimes results in distinct phenotypes. For instance, although genetic elimination of the upstream kinase MST1/2 in the mouse liver [26–28] and intestine [38] has been associated with tumorigenesis, deletion of the downstream kinase LATS1/2 in the liver [36, 37], kidney [39], and breast [40] does not lead to cancer development in mice, presenting a compelling contrast in the observed outcomes [41]. Noncanonical functions of these molecules, apart from those of the Hippo pathway, may cause this phenotypic variation. However, the intensity of the Hippo pathway inhibition may also affect the resulting phenotype. Indeed, MST1/2 has been shown to be redundant for proper YAP/TAZ regulation, whereas LATS1/2 is indispensable in certain cell lines [40], suggesting that the inhibition of the Hippo pathway is more severe upon deletion of LATS1/2 than upon deletion of MST1/2. Therefore, the biological functions of the Hippo pathway are context dependent, and the timing and intensity of Hippo pathway inhibition affect the overall tissue phenotype outcome. This complexity and superficial discrepancy in the phenotypic variation among Hippo pathway-mutant animals pose a major challenge in the field.

Although the exact mechanisms underlying for the complex roles of the Hippo pathway in different biological contexts have not been elucidated, recent studies revealed cell-type-dependent and non-cell-autonomous functions of the Hippo pathway, in addition to the classically well-characterized cell-autonomous functions of the Hippo pathway. These emerging roles of the Hippo pathway in multicellular organisms, wherein different cell types coordinately maintain tissue homeostasis by interacting with each other, may help in understanding the context-dependent functions of this pathway.

#### Context-dependent functions of the Hippo pathway

Recently, it has been revealed that the phenotypes from Hippo pathway mutations differed between cell types. Although the loss of LATS1/2 or activation of YAP/TAZ promotes cell proliferation in most cell lines, LATS1/2 deletion inhibits the growth of murine colon adenocarcinoma MC38 cells owing to the induction of the cellular growth suppressor WNT1-inducible signaling pathway protein 2 (WISP2) and coiled-coil domain containing 80 (CCDC80) [42]. Similarly, YAP activation leads to excessive accumulation of reactive oxygen species by downregulating the antioxidant enzyme glutathione peroxidase 2 (GPX2), inhibiting the growth of lung squamous cell carcinoma [43]. Furthermore, loss of LATS1/2 or overexpression of YAP is sufficient to reprogram cancer stem cells to attenuate Wnt signaling, thereby suppressing tumor growth in organoids, patient-derived xenografts, and mouse models of primary and metastatic colorectal cancer (CRC) [44]. Therefore, although the Hippo pathway is generally considered to suppress cell growth, it can also promote cell proliferation and survival in some cases, and the biological functions of the Hippo pathway are context dependent.

This cell type-dependent phenotypic difference could result from differences in the histone modifications and chromatin accessibility between different cell types. Recently, it was shown that YAP modulates chromatin accessibility [45, 46], and that YAP/TAZ in complex with TEAD family transcription factors mainly bind to distal enhancer regions to regulate gene expression [47–49]. In fact, 91% of the YAP/TAZ/TEAD4 complex was identified in the enhancer region, whereas only a small fraction (3.6%) was localized to the promoter region [49]. Given that enhancers are genetic elements that confer cell type-specific gene expression patterns [50], the target gene expression regulated by YAP/TAZ, and thus the functional output of the Hippo pathway, can vary widely across cell types, contributing to the diverse cellular phenotypes regulated by the Hippo pathway.

**Fig. 2:**
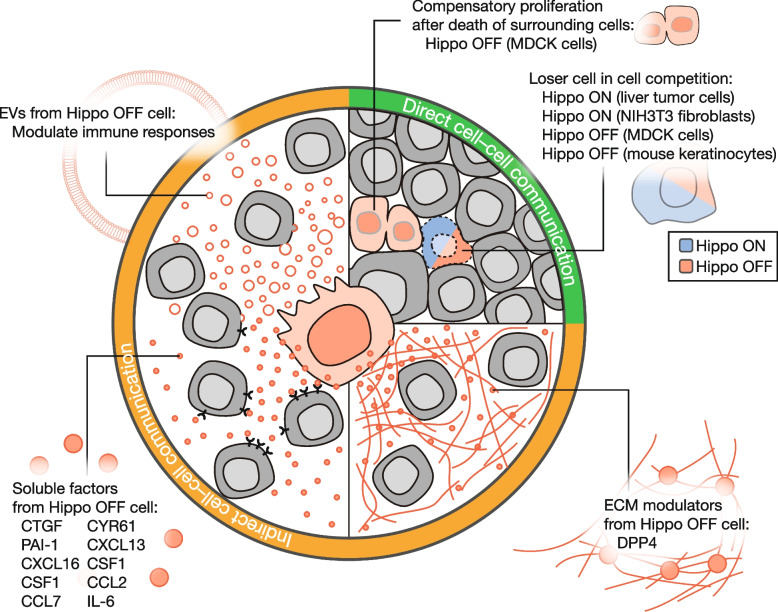
Schematic representation of the cell–cell communication regulated by the Hippo pathway. Intercellular communication can be categorized as direct or indirect. Direct cell–cell communication is mediated by direct interactions between cells, whereas indirect cell–cell communication involves soluble factors, extracellular vesicles (EVs), or the extracellular matrix (ECM). Examples include the molecules regulated by the Hippo pathway

Another plausible mechanism underlying the context-dependent functions of the Hippo pathway in tissue homeostasis is cell–cell communication induced by the Hippo pathway. Owing to its inhibitory role in cell proliferation, the Hippo pathway is believed to act as a tumor suppressor, and suppression of the Hippo pathway may promote tumor progression. Consistent with the growth inhibitory effect of LATS1/2, the deletion of LATS1/2 promoted cancer cell growth *in vitro*. However, a previous study revealed that the inhibition of the Hippo pathway in cancer cells unexpectedly suppressed tumor growth in mice [51]. LATS1/2 deletion in cancer cells also induces strong immune responses, overwhelming any growth advantage gained by the loss of LATS1/2 and leading to strong inhibition of tumor growth *in vivo*. Another study showed that LATS1/2 deletion in tumor-surrounding cells eliminated cancer cells through cell competition in mice [52]. Therefore, the survival or death of cancer cells depends on competing Hippo signaling in the tumor and surrounding tissues. These results imply that the Hippo intracellular signaling pathway not only affects the fate of the given cells but also regulates the surrounding cells in a non-cell-autonomous manner, inducing diverse cell–cell communication. The characteristics of the Hippo pathway in regulating cell–cell communication add an extra level of complexity and diverse regulatory options to the Hippo pathway for the maintenance of tissue homeostasis, which may be closely associated with the context-dependent functions of this pathway. Herein, we highlight a recent understanding of the mechanisms by which the Hippo pathway modulates the surrounding environment through cell–cell communication (Fig. 2).

### The Hippo pathway in direct cell–cell communication

The number of cells in tissues is tightly controlled by cell proliferation and cell death, and dysregulation of this process can lead to pathological conditions such as cancer or hypoplasia. Both cell competition and compensatory proliferation contribute to the maintenance of tissue homeostasis.

#### Cell competition

Morata and Ripoll introduced the concept of cell competition [53]. Cell competition is defined as a selection process that occurs within a tissue to eliminate cells with low fitness through interactions between cells of different fitness [54–58]. While much of our understanding of cell competition comes from work performed in *Drosophila*, recent studies have shed light on its role in mammalian development [55, 59], especially during the early postimplantation stages [60]. The pluripotent cell population known as epiblasts is initially established in preimplantation embryos, and its integrity is crucial for appropriate embryonic development. Hashimoto et al. revealed that TEAD transcriptional activity is essential for robust expression of pluripotency factors and exhibits variability in the developing epiblast. Cells with low TEAD activity undergo elimination through cell competition within the epiblast, eliminating unspecified cells and ensuring proper epiblast organization [61]. This finding suggests that the induction of pluripotency by the Hippo pathway and the removal of unspecified cells through cell competition are essential for generating epiblasts with naive pluripotency.

Other examples of cell competition induced by the Hippo pathway have been studied in both *in vitro* cell culture as well as *in vivo* mouse models. Mouse NIH3T3 fibroblasts with reduced TEAD activity are preferentially eliminated by apoptosis as loser cells, whereas those with increased TEAD activity became winner to eliminate neighboring normal cells [62]. In contrast, Madin-Darby canine kidney (MDCK) cells overexpressing an active mutant form of YAP were eliminated from the culture monolayer as loser cells via apical extrusion [63]. Similarly, mouse keratinocytes overexpressing an active mutant form of YAP or cells with activated endogenous YAP due to neurofibromatosis type 2 (NF2) knockdown were defeated in *in vitro* competition with control cells experiencing apical extrusion [64]. Although the exact mechanisms underlying these cell type-specific effects of YAP activation on cell competition remain unclear, YAP-overexpressing keratinocytes show reduced cell–matrix adhesion due to the defective expression of adhesion molecules like fibronectin-1. Indeed, the MOB1A- and MOB1B-deficient epidermis, and thus the YAP-activated epidermis, exhibits decreased type XVII collagen expression and cannot be successfully engrafted onto donor mice [64]. Therefore, it is possible that a difference in the ability of cells to adhere to the surface of their surrounding matrix environment may cause winner–loser switching in cell competition in different contexts and cell types.

#### Compensatory proliferation

To maintain cell numbers in the tissue, a cell elimination process stimulates the proliferation of neighboring cells. This process is known as compensatory proliferation. Although only a limited number of neighboring cells must divide to compensate for cell loss, the mechanisms that select cells for division have not been fully elucidated. A recent study using an MDCK monolayer cell culture system revealed that the inhomogeneous activity of YAP-mediated mechanotransduction in neighboring cells determines cell division for compensatory proliferation [65]. This inhomogeneity in YAP activity arises from the nonuniform distribution of nuclear size and the mechanical force exerted on neighboring cells. Upon the induction of apoptosis, only the surrounding cells with a large nucleus that experienced a large deformation underwent cell division. Although the mechanisms by which nuclear translocation of YAP leads to cell cycle progression in the context of apoptosis-induced proliferation remain to be elucidated, these results highlight that the heterogeneity of YAP activity and force propagation among neighboring cells plays a causal role in compensatory proliferation.

### The Hippo pathway in indirect cell–cell communication

Hippo pathway responds to soluble cues and mechanical stimuli from the cellular microenvironment. Receptors such as G protein-coupled receptors (GPCRs) and adherence complexes within the plasma membrane sense these soluble cues to regulate the activity of the Hippo pathway [66–70]. The Hippo pathway integrates a wide range of mechanical signals, including shear stress, contractile forces, and extracellular matrix (ECM) stiffness, and translates these signals into cell-specific programs by regulating gene expression [69, 71, 72]. Therefore, the Hippo pathway acts as a nexus and an integrator of multiple signals from the cellular microenvironment, determining multiple aspects of cell fate. Additionally, recent studies have shown that the activity of the Hippo pathway within cells regulates the release of soluble factors, extracellular vesicles, and the ECM to determine the fate of neighboring cells.

#### Soluble factors

Previous studies have identified connective tissue growth factor (CTGF) and cysteine-rich angiogenesis-inducing factor 61 (CYR61) as bona fide YAP/TAZ target genes [73, 74]. CTGF and CYR61 belong to a protein family of cell communication networks (CCN) and coordinate cell functions by interacting with ECM structural proteins, cell-surface receptors, proteases, and hormones [75]. These proteins regulate cellular functions in a cell-autonomous and non-cell-autonomous manner. A previous study revealed that YAP-mediated expression of CYR61 in proliferating cells enhances the survival of neighboring cells [76]. In the context of pluripotency induction from mouse somatic cells, YAP inhibits pluripotency induction in a cell-autonomous manner yet promotes pluripotency induction of neighboring cells non-cell autonomously by inducing the expression of secreted factors, mainly CYR61 [77]. These results imply that the well-characterized biological outputs of the Hippo pathway, including cell survival and stem cell maintenance, are mediated by both cell-autonomous and non-cell-autonomous mechanisms involving soluble factors.

In addition, Hippo pathway-regulated soluble factors play important roles in inflammation and immune responses. Liver injury in both mice and humans elevates the YAP/TAZ levels to induce CYR61 expression in hepatocytes. This process contributes to macrophage infiltration and subsequent liver inflammation and fibrosis [78]. Another study showed that the activation of YAP in tumor-initiating cells (TICs) recruits TIC-associated macrophages via the induction of C-C motif chemokine ligand 2 (CCL2) and colony-stimulating factor 1 (CSF1), suppressing the immune clearance of TICs to promote tumorigenesis [79]. Intriguingly, this property of the Hippo pathway in regulating cytokines, along with its regulation by them, contributes to establishing a feed-forward loop to confer a robust pathological response for disease progression. Stromal fibroblast-derived periostin activates YAP/TAZ in colon cancer cells. This induction leads to interleukin (IL)-6 production from cancer cells, which, in turn, activates and further induces periostin expression in fibroblasts [80]. Therefore, the Hippo pathway confers periostin- and IL-6-mediated interactions between tumors and the surrounding stroma, establishing a feed-forward loop to promote colorectal tumorigenesis. These observations suggest that the Hippo pathway regulates a wide range of cytokines and chemokines to induce cell–immune cell communication, enabling various tissue responses in different biological contexts. Indeed, proteome profiling analysis revealed that YAP overexpression promotes the expression and secretion of numerous paracrine-acting factors, including plasminogen activator inhibitor-1 (PAI-1), C-X-C motif chemokine ligand 13 (CXCL13), and CXCL16 during hepatocarcinogenesis [81]. Collectively, soluble factors regulated by the Hippo pathway affect the fate of the surrounding cells and modulate immune responses, inducing a multicellular response that connects intracellular events with tissue responses.

#### Extracellular vesicles

Extracellular vesicles (EVs) are small particles naturally released from various cell types. These vesicles contain a diverse array of cellular elements including proteins, nucleic acids, lipids, and metabolites. Their composition enables intercellular communication that plays a crucial role in various biological processes [82–86]. Recent studies have revealed mutual regulation between the Hippo pathway and EVs.

The effect of EVs on the regulation of the Hippo pathway has been extensively studied and summarized elsewhere [87]. For instance, in a mouse model of CRC liver metastasis, EVs derived from hepatocytes in fatty liver transfer miRNAs that suppress LATS2 expression and induce YAP activation. These EVs promote cancer cell growth and creating an immunosuppressive microenvironment marked by M2 macrophage infiltration through CYR61 production [88]. In contrast, the Hippo pathway also regulates EV biogenesis. One study demonstrated that CD47-enriched EVs are released from hepatocytes in a YAP-dependent manner, inhibiting dendritic cell activation and ameliorating hepatic ischemia-reperfusion injury [89]. Another study showed that the 5-methylcytosine modification of YAP mRNA stabilized it and increased EV release in lung adenocarcinoma [90]. This effect is driven by two YAP-dependent transcription factors, Mycn and SRY-box transcription factor 10 (SOX10), which activate the transcription of several downstream genes promoting EV release. Although the molecular mechanisms underlying EV biogenesis have only begun to be elucidated, future studies clarifying the molecular link between the Hippo pathway and EV biogenesis will expand our understanding of how environmental cues are sensed and integrated to modulate EV composition and quantity, thereby broadening EV biology.

#### Extracellular matrix

The Hippo pathway is known to respond to ECM stiffness [69, 91–93]. For instance, the Ras-related GTPase RAP2A/B/C, which functions as a binding partner of the Hippo upstream kinases MAP4K1/2/3/4/5/6/7, is activated under low stiffness conditions to stimulate the Hippo pathway, transducing signals from ECM stiffness to control gene expression via YAP/TAZ [72]. Additionally, emerging evidence in recent years suggests that the Hippo pathway is an important modulator of the ECM. During cardiac development, LATS1/2 induce cardiac fibroblast differentiation from epicardial progenitors while concurrently controlling ECM composition and vascular remodeling [94]. Cells lacking LATS1/2 exhibit developmental arrest characterized by the sustained presence of epicardial markers and elevated expression of YAP targets, including dipeptidyl peptidase 4 (DPP4), a protease involved in ECM remodeling. Thus, the Hippo pathway suppresses a gene program coordinating ECM composition, thereby affecting crucial aspects of vessel development, such as endothelial cell proliferation, migration, and vessel branching. Similarly, the Hippo pathway also regulate the differentiation and activation of fibroblasts in different biological contexts; LATS1/2-deficient adipocytes accelerate fibrosis by dedifferentiating into DPP4^+^ progenitor cells and, upon transforming growth factor β (TGFβ) stimulation, transforming into DPP4^−^ myofibroblasts [95]. In the context of cancer, YAP in cancer-associated fibroblasts (CAFs) increases matrix stiffness, facilitating cancer cell invasion and promoting angiogenesis. YAP plays a pivotal role in governing the expression of various cytoskeletal regulators such as anillin (ANLN), diaphanous-related formin 3 (DIAPH3), and myosin light chain 9 (MYL9) [96]. YAP activation is further intensified in response to matrix stiffening, creating a feed-forward, self-reinforcing loop that contributes to sustained CAF phenotype and cancer progression.

Collectively, the ability of the Hippo pathway to regulate cell–cell communication via direct cell–cell interactions, soluble factors, EVs, and ECM offers multiple options for this pathway to control diverse biological processes. Therefore, the Hippo pathway not only determines the fate of a given cell but also induces multicellular responses involving tissue-resident cells as well as infiltrating immune cells. Additionally, the responsiveness of the Hippo pathway to various extracellular signals, including soluble factors and ECM stiffness, enables the establishment of a feedback or feed-forward loop, conferring robust homeostatic regulation in tissue physiology or a vicious cycle in disease progression. This contributes to the involvement of the Hippo pathway in a broad range of life events, including organ development, regeneration, aging, and cancer. In the next section, we highlight the importance of cell–cell communication mediated by the Hippo pathway in development and regeneration.

### Hippo pathway-mediated intercellular communications during development

**Fig. 3:**
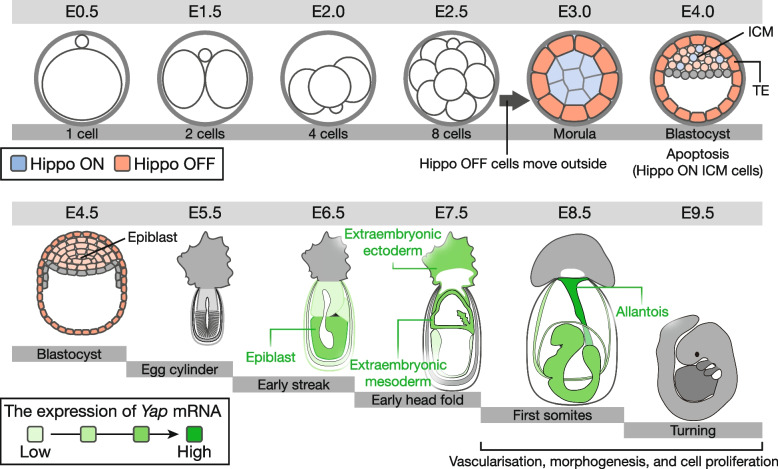
Hippo pathway-mediated intercellular communications during development. The Hippo pathway contributes to cell differentiation from the eight-cell stage by being activated in cells with pluripotent inner cell mass (ICM) but not in cells of multipotent trophectoderm (TE) lineages. Subsequently, cells with high Hippo pathway activity are eliminated by apoptosis, owing to cell competitive mechanisms at the blastocyst stage, selecting highly pluripotent cells in the ICM. Between E6.5 and E8.5, YAP mRNA expression remains high, although its precise functions remain to be elucidated. YAP activity is associated with vascularization, morphogenesis, cell proliferation, and organogenesis in multiple organs

The Hippo pathway is distinct from other developmental signaling pathways in terms of its ability to respond to various signals such as hormones, ECM, mechanical cues, energy status, and cellular stress, rather than specific morphogens. This pathway integrates these diverse inputs and regulates cellular events that are crucial for cell growth, differentiation, overall tissue development, and homeostasis (Fig. 3).

In embryos at the 8- to 16-cell stage, active TEAD4 induces caudal-type homeobox 2 (CDX2) expression and promotes trophectoderm (TE) development in extracellular cells [97, 98]. In contrast, TEAD4 activity is suppressed inside cells of the inner cell mass (ICM) lineage by cell–cell contact-induced YAP inhibition by LATS [99]. In the early blastocyst ICM, high Hippo signaling activity keeps YAP out of the nucleus, rendering TEAD transcription factors inactive [99]. Temporal reduction of LATS1/2 results in nuclear accumulation of YAP within inner cells, leading to an abnormal morphology of the ICM with collapsed clumps of cells and inducing a shift in cell fate toward a TE-like lineage [100]. Consistently, inhibiting the upstream regulator NF2 using dominant-negative mRNA induces nuclear translocation of YAP and ectopic expression of CDX2 inside cells, adopting a TE fate and failing to form an ICM [101]. The activity of the Hippo pathway, which is regulated by cell–cell contact, is crucial for the induction of ICM/TE lineage specification.

At the mid-blastocyst stage, nuclear YAP/TEAD activity and expression of pluripotency factors are highly heterogeneous in the ICM. During epiblast formation, YAP gradually accumulates in the nucleus, activating TEAD transcriptional programs. Elevated TEAD transcriptional activity induces the expression of pluripotency factors that facilitate the formation of epiblasts. During this step, cells compete to eliminate cells with low TEAD activity through apoptosis, thereby contributing to the removal of less pluripotent cells. This process ensures the generation of high-quality epiblasts characterized by a state of naïve pluripotency [102, 103].

Following the preimplantation stage, embryonic development enters the gastrulation and neurulation stage from embryonic days 6 (E6) to E9 in mice. *Yap* mRNA is broadly distributed in the trophectoderm and epiblast derivatives from E6.5 to E8.5 yet demonstrates stage- and region-specific domains with relatively strong expression [104]. Mouse embryos lacking YAP arrest development around E8.5 and display defects in yolk sac vascular development, chorioallantoic fusion, and embryonic axis elongation [104]. Despite a clear indication of appropriate patterning and lineage specifications, YAP-deficient embryos display developmental abnormalities. This suggests that at this stage of embryogenesis, YAP is required for morphogenetic movement or the maintenance of proper cell numbers, rather than lineage specification. From this point onwards, mouse development (E9−) undergoes organogenesis. During this period, the Hippo pathway is considered to play an important role in cell survival, migration, and 3D structure formation [105]. However, the involvement of Hippo pathway-mediated intercellular communication in embryonic development after gastrulation remains unclear.

### Hippo pathway-mediated intercellular communications in regeneration

**Fig. 4:**
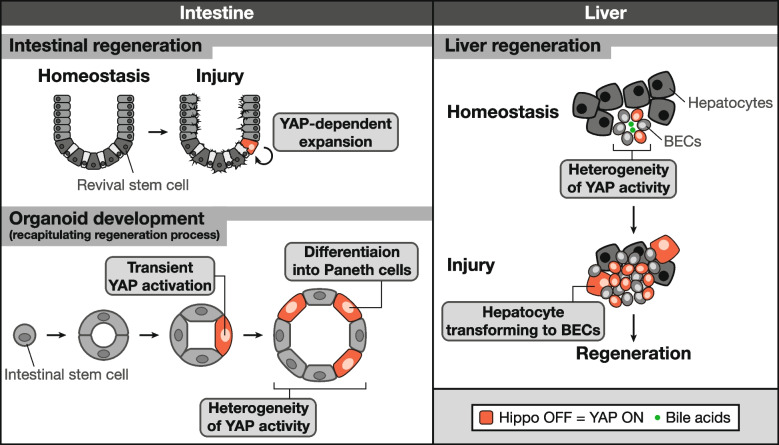
Hippo pathway-mediated intercellular communications in regeneration. In the intestine, revival stem cells are infrequent under normal conditions; however, they undergo YAP-dependent expansion to facilitate intestinal regeneration in response to injury. During intestinal organoid development from a single cell, YAP activation exhibits heterogeneity within intestinal spheres. This heterogeneity is essential for the activation of Notch–Dll1, thereby promoting the formation of Paneth cells. In a normal liver, biliary epithelial cells (BECs) exhibit significant diversity in YAP activity, illustrating the dynamic cellular state and plasticity of BECs during homeostasis. In the event of liver injury, YAP-active BECs increase in response to bile acids, which is crucial for BEC survival and plays a pivotal role in reprogramming hepatocytes into biliary progenitors for the regenerative response

The Hippo pathway plays an essential role in regeneration, particularly in the intestine [106], liver [107–109], heart [110–112], and skin [68], where, in most cases, transient YAP/TAZ activation promotes regenerative tissue repair. However, achieving regeneration after injury by manipulating the Hippo pathway is not simple; sustained YAP/TAZ activation in the entire tissue may result in ectopic cell proliferation, ultimately leading to hyperplasia or neoplasia. Recent studies have revealed that heterogeneous, rather than homogenous, YAP activation within tissues is important for regeneration in several contexts (Fig. 4).

For instance, single-cell RNA sequencing has identified a damage-induced quiescent cell type called revival stem cells in the mouse intestinal epithelium [113]. These stem cells are extremely rare under normal conditions but undergo YAP-dependent transient expansion following damage, giving rise to all major cell types of the intestine to regenerate the intestinal epithelium. Using an *in vitro* organoid system that mimics the regenerative process, another study revealed that transient activation of YAP, followed by variability in YAP subcellular localization within the intestinal sphere, is crucial for Paneth cell differentiation [114]. The induction of delta-like protein 1 (Dll1), a YAP target gene, within YAP-activated cells leads to the activation of Notch signaling in neighboring cells via lateral inhibition, driving Paneth cell formation to constitute the key niche to support intestinal stem cells. Homogeneous activation or suppression of YAP failed to activate Notch–Dll1 signaling, thereby abolishing Paneth cell differentiation and organoid budding. Therefore, cell–cell communication induced by the heterogeneous Hippo pathway status among neighboring cells is critical for proper intestinal regeneration and tissue organization.

Similar heterogeneity in YAP activity was observed during liver regeneration. In the liver, hepatocytes and BECs repair tissues following injury. BECs contribute to liver repopulation and exhibit substantial plasticity under certain conditions [115, 116]. A study using single-cell RNA sequences revealed substantial heterogeneity of BECs in the normal liver, reflecting the dynamic activation of a YAP-dependent transcriptional program [117]. This transcriptional signature delineates a dynamic cellular state during homeostasis and exhibits a high responsiveness to injury, highlighting the plasticity of BECs. During liver injury, YAP-active BECs increase in response to bile acids, which are necessary for BEC survival and crucial for hepatocyte reprogramming into biliary progenitors for the regenerative response. However, it remains to be elucidated how this heterogeneity in the YAP activation status in BECs contributes to the efficient induction of liver regeneration.

## Conclusions

Numerous studies have focused on the cell-autonomous functions of the Hippo pathway, which mediates the intracellular signaling cascade to regulate gene expression. In addition to these previously well-characterized functions of the Hippo pathway, recent studies have revealed that cells can change the activity of their own Hippo pathway to control the fate of surrounding cells either directly (cell–cell interactions) or indirectly (interactions via soluble factors, EVs, or ECM) via cell–cell communication. Non-cell-autonomous functions of the Hippo pathway have emerged as crucial regulators of development, regeneration, and cancer development.

Although the Hippo pathway was initially identified as a regulator of organ size, previous studies have suggested that it may not necessarily regulate normal organ growth. For instance, the conditional deletion of YAP/TAZ at the embryonic stage of liver development does not interfere with hepatocyte proliferation or liver growth [118, 119]. Additionally, a recent study revealed that numerous genes targeted by YAP/TAZ are associated with ectopic rather than normal growth [120]. These results imply that the Hippo pathway does not dictate when cells proliferate or undergo apoptosis during normal development. Thus, the classic Hippo mutant overgrowth phenotype may represent abnormal activation of the genetic program that facilitates ectopic overgrowth [120]. Consistently, YAP-deficient embryos demonstrate appropriate patterning and lineage specification at E8.5 [104], suggesting that defects in the cell-autonomous functions of the Hippo pathway may not be the primary reason for the embryonic lethality of YAP-null mice. However, despite clear indications for normal cellular differentiation, YAP-deficient embryos exhibit abnormal morphogenesis and development. Therefore, one may speculate that some aspects of the physiological functions of the Hippo pathway during embryogenesis are to maintain high-quality cells and facilitate the morphogenetic movement of the cells through cell–cell communication. As highlighted in this review, the non-cell-autonomous functions of the Hippo pathway in mammals have been elucidated by taking advantage of recent developments in single-cell analysis and/or the introduction of sporadic Hippo mutations in tissues. Notably, recent advances in the genetic tracing of cell–cell contacts using the synthetic Notch (synNotch) system, which utilizes artificial Notch ligand–receptor interaction to induce gene expression upon cell–cell contact [121, 122], and the photo-isolation chemistry (PIC) system, which enables the isolation of transcriptome profiles from photo-irradiated regions of interest [123], have revealed an unexpected broad cell–cell interaction history during development. Future studies clarifying the role of the Hippo pathway in these intercellular communications during development and regeneration may not only contribute to a better understanding of this pathway but also open new avenues for future therapeutic strategies targeting the Hippo pathway.

## Data Availability

Not applicable

## Abbreviations

MST1/2: Mammalian STE20-like kinase 1/2
MAP4K1/2/3/4/5/6/7: Mitogen-activated protein kinase kinase kinase kinase 1/2/3/4/5/6/7
LATS1/2: Large tumor suppressor homolog
SAV1: Salvador family WW domain containing protein 1
MOB1A/B: MOB kinase activator 1A/B
YAP: Yes-associated protein
TAZ: Transcriptional coactivator with PDZ-binding motif
TEAD1/2/3/4: TEA domain transcriptional factor 1/2/3/4
BECs: Biliary epithelial cells
MC38: Murine colon adenocarcinoma
WISP2: WNT1-inducible signaling pathway protein 2
CCDC80: Coiled-coil domain containing 80
GPX2: Glutathione peroxidase 2
CRC: Colorectal cancer
MDCK: Madin-Darby canine kidney
NF2: Neurofibromatosis type 2
GPCRs: G protein-coupled receptors
ECM: Extracellular matrix
EVs: Extracellular vesicles
CTGF: Connective tissue growth factor
CYR61: Cysteine-rich angiogenesis-inducing factor 61
CCN: Cell communication network
TICs: Tumor-initiating cells
CCL2: C-C motif chemokine ligand 2
CSF1: Colony-stimulating factor 1
IL: Interleukin
PAI-1: Plasminogen activator inhibitor 1
CXCL13: C-X-C motif chemokine ligand 13
SOX10: SRY-box transcription factor 10
DPP4: Dipeptidyl peptidase 4
TGFb: Transforming growth factor b
CAFs: Cancer-associated fibroblasts
ANLN: Anillin
DIAPH3: Diaphanous-related formin 3
MYL9: Myosin light chain 9
CDX2: Caudal type homeobox 2
TE: Trophectoderm
ICM: Inner cell mass
TGFβ: Transforming growth factor β
E6: Embryonic days 6
synNotch: Synthetic Notch
PIC: Photo-isolation chemistry
DLL1: Delta-like protein 1
